# Supplementary material for: Plastid phylogenomics and cytonuclear discordance in Rubioideae, Rubiaceae
Source: PLoS One. 2024 May 20;19(5):e0302365. doi: 10.1371/journal.pone.0302365 (PMC11104678; doi:10.1371/journal.pone.0302365)
Supplement: S1 Table — (PDF) [file pone.0302365.s001.pdf]

S1 Table. Taxa, voucher/source, ENA/GenBank, assembly, and sequencing information for sequences used in this study.

Taxa with fully assembled plastomes are indicated in grey.

Lab ID	Species	Family (Subfamily)	Tribe (Rubiaceae only)	Collection/Source	Year	Collection locality	Assembly strategy*
	Alstonia scholaris (L.) R.Br.	Apocynaceae		Antonelli et al. (2021)/PAFTOL			RG-only
	Chironia baccifera L.	Gentianaceae		Antonelli et al. (2021)/PAFTOL			RG-only
	Geniostoma borbonicum (Lam.) Spreng.	Loganiaceae		Antonelli et al. (2021)/PAFTOL			RG+de novo
BD003	Acranthera grandiflora Bedd.	Rubiaceae	Coptosapelteae	J. Klackenberg & R. Lundin 541 (S)	1982	India, Tamil Nadu	RG+de novo
AY008	Coptosapelta diffusa (Champ. ex Benth.) Steenis	Rubiaceae	Coptosapelteae	Steward et al 594 (S)	1931	China	RG+de novo
AZ040	Luculia pinceana Hook.	Rubiaceae	Luculieae	Thin et al. 3061 (AAU)			de novo
DE068	Cinchona calisaya Wedd.	Rubiaceae (Cinchonoideae)	Cinchoneae	Razafimandim. & Razafimanant. 471 (UPS)	2002	Madagascar	RG+de novo
CC071	Deppea grandiflora Schltdl.	Rubiaceae (Cinchonoideae)	Hamelieae	Marino Rosas R. 1015 (P)			RG-only
DE066	Paracorynanthe antankarana Capuron ex J.-F.Leroy	Rubiaceae (Cinchonoideae)	Hymenodictyeae	B. Bremer et al. 5156 (S)	2008	Madagascar	RG+de novo
	Corynanthe pachyceras K.Schum.	Rubiaceae (Cinchonoideae)	Naucleeae	Antonelli et al. (2021)/PAFTOL			RG-only
AG098	Rondeletia intermixta Britton	Rubiaceae (Cinchonoideae)	Rondeletieae	Rova et al. 2245 (GB)	1995	Cuba	RG+de novo
AP035	Airosperma vanuense S.P.Darwin	Rubiaceae (Ixoroideae)	Airospermeae	Smith 8214 (P)	1953	Fiji	RG+de novo
	Razafimandimbisonia minor (Baill.) Kainul. & B.Bremer	Rubiaceae (Ixoroideae)	Alberteae	Antonelli et al. (2021)/PAFTOL			RG-only
BE034	Wendlandia dasythyrsa Miq.	Rubiaceae (Ixoroideae)	Augusteae	Christensen, H. M. 460 (AAU)	1993	Malaysia	RG+de novo
BD032	Alseis lugonis L.Andersson	Rubiaceae (Ixoroideae)	Condamineeae	Bremer et al. 3353 (UPS)	1995	Ecuador	RG-only
	Emmenopterys henryi Oliv.	Rubiaceae (Ixoroideae)	Condamineeae	Antonelli et al. (2021)/PAFTOL			RG-only
CX014	Ixora javanica (Blume) DC.	Rubiaceae (Ixoroideae)	Ixoreae	Puff 000512-1/2 (WU)		Vietnam	RG+de novo
	Jackiopsis ornata (Wall.) Ridsdale	Rubiaceae (Ixoroideae)	Jackieae	Antonelli et al. (2021)/PAFTOL			RG-only
	Heinsia crinita (Wennberg) G.Taylor	Rubiaceae (Ixoroideae)	Mussaendeae	Antonelli et al. (2021)/PAFTOL			RG-only
AV015	Molopanthera paniculata Turcz.	Rubiaceae (Ixoroideae)	Posoquerieae	Williams & Assis 6861 (S)	1945	Brazil	de novo
CG080	Sabicea marojejyensis Razafim. & J.S.Mill.	Rubiaceae (Ixoroideae)	Sabiceeae	Bremer et al. 5293 (S)	2008	Madagascar	de novo
	Cyclophyllum barbatum (G.Forst.) N.Hallé & J.Florence	Rubiaceae (Ixoroideae)	Vanguerieae	Antonelli et al. (2021)/PAFTOL			RG+de novo
CA098	Glionnetia sericea (Baker) Tirveng.	Rubiaceae (Ixoroideae)		Beaver 3 (S)	2009	Seychelles	de novo
DE070	Anthospermum thymoides Baker	Rubiaceae (Rubioideae)	Anthospermeae	Thureborn et al. 33 (S)	2017	Madagascar	RG+de novo
DE067	Carpacoce spermacocea (Rchb. ex Spreng.) Sond.	Rubiaceae (Rubioideae)	Anthospermeae	Bremer et al. 4385 (UPS)	2002	South Africa	RG+de novo
	Coprosma longifolia A.Gray	Rubiaceae (Rubioideae)	Anthospermeae	Antonelli et al. (2021)/PAFTOL			RG+de novo
CY012	Coprosma rhamnoides A.Cunn.	Rubiaceae (Rubioideae)	Anthospermeae	Tibell NZ46 (UPS)	1980	New Zealand	RG+de novo
DE081	Durringtonia paludosa R.J.F.Hend. & Guymer	Rubiaceae (Rubioideae)	Anthospermeae	Thompson 18m11 (BRI)	2001	Australia, Queensland	RG+de novo
CX098	Galopina aspera (Eckl. & Zeyh.) Walp.	Rubiaceae (Rubioideae)	Anthospermeae	Phillipson 1461 (UPS)	1986	South Africa	RG+de novo
CX099	Leptostigma pilosum (Benth.) Fosberg	Rubiaceae (Rubioideae)	Anthospermeae	Asplund 7171 (UPS)	1939	Ecuador	RG+de novo
CY003	Nertera dichondrifolia (A.Cunn.) Hook.f.	Rubiaceae (Rubioideae)	Anthospermeae	Tibell NZ119 (UPS)	1980	New Zealand	de novo
DE075	Normandia neocaledonica Hook.f.	Rubiaceae (Rubioideae)	Anthospermeae	Selling 125b (S)	1949	New Caledonia	RG+de novo
CY089	Opercularia volubilis R.Br. ex Benth.	Rubiaceae (Rubioideae)	Anthospermeae	B.J. Lepschi & B.A. Fuhrer BJL 3671 (P)	1997	Australia	de novo
CY051	Phyllis nobla L.	Rubiaceae (Rubioideae)	Anthospermeae	Wikström et al. 76 (S)	1994	Tenerife	de novo
DE074	Pomax umbellata (Gaertn.) Sol. ex A.Rich.	Rubiaceae (Rubioideae)	Anthospermeae	Halford Q9744 (BRI)	2009	Australia	RG+de novo
BL040	Argostemma elatostemma Hook.f.	Rubiaceae (Rubioideae)	Argostemmateae	Bremer and Bremer 1722 (S)	1979	Malaysia, Sarawak	RG+de novo
BU095	Clarkella nana (Edgew.) Hook.f.	Rubiaceae (Rubioideae)	Argostemmateae	Maxwell 02-252 (MO)			RG-only
CH091	Mouretia larsenii Tange	Rubiaceae (Rubioideae)	Argostemmateae	van Beusekom 4743 (P)	1972	Thailand	de novo
CD007	Mycetia bracteata Hutch.	Rubiaceae (Rubioideae)	Argostemmateae	Steward & Cheo 1105 (S)	1933	China	de novo
	Mycetia Reinw.	Rubiaceae (Rubioideae)	Argostemmateae	Antonelli et al. (2021)/PAFTOL			RG-only
BK078	Neohymenopogon parasiticus (Wall.) Bennet	Rubiaceae (Rubioideae)	Argostemmateae	Bremer 2743 (UPS)		Cult.	RG+de novo
BD002	Colletoecema dewevrei (De Wild.) E.M.A.Petit	Rubiaceae (Rubioideae)	Colletoecemateae	Lisowski 47195 (K)	1977	Congo	NA
BA061	Coccocypselum condalia Pers.	Rubiaceae (Rubioideae)	Coussareeae	Persson, C. & Gustafsson, C. 246 (GB)	1996	Bolivia	RG+de novo

S1 Table. Taxa, voucher/source, ENA/GenBank, assembly, and sequencing information for sequences used in this study.

Taxa with fully assembled plastomes are indicated in grey.

Lab ID	Species	Family (Subfamily)	Tribe (Rubiaceae only)	Collection/Source	Year	Collection locality	Assembly strategy*
DA042	Cruckshanksia pumila Clos in C.Gay	Rubiaceae (Rubioideae)	Coussareeae	Taylor et al. 10679 (MO)	1991	Chile	RG+de novo
DB007	Faramea multiflora A.Rich. ex DC.	Rubiaceae (Rubioideae)	Coussareeae	Salino 3825 (MO)	1997	Brazil	RG+de novo
	Craterispermum Benth. 2	Rubiaceae (Rubioideae)	Craterispermeae	Antonelli et al. (2021)/PAFTOL			RG-only
CK013	Craterispermum Benth. 1	Rubiaceae (Rubioideae)	Craterispermeae	Razafimandimbison et al. 1168 (S)	2011	Madagascar	RG+de novo
CL038	Craterispermum schweinfurthii Hiern	Rubiaceae (Rubioideae)	Craterispermeae	J. D & E. G. Chapman 9364 (UPS)	1988	Malawi	RG+de novo
BZ084	Cyanoneuron cyaneum (Hallier f.) Tange	Rubiaceae (Rubioideae)	Cyanoneuroneae	Bogner 1457 (L)		Borneo, Indonesia	RG+de novo
	Cyanoneuron pedunculatum Tange	Rubiaceae (Rubioideae)	Cyanoneuroneae	Antonelli et al. (2021)/PAFTOL			RG-only
DB070	Danais nigra Homolle	Rubiaceae (Rubioideae)	Danaideae	N. S. Rasoanaivo & A. J. Tahinarivony 69 (S)	2012	Madagascar	RG+de novo
DB077	Payera decaryi (Homolle) Buchner & Puff	Rubiaceae (Rubioideae)	Danaideae	Krüger & Razafimandimbison 74 (S)	2010	Madagascar	de novo
DC094	Schismatoclada marojejyensis Humbert	Rubiaceae (Rubioideae)	Danaideae	Bremer et al. 5309 (S)	2008	Madagascar	de novo
BC005	Dunnia sinensis Tutcher	Rubiaceae (Rubioideae)	Dunnieae	Xinhui 16 (in Ge et al, 2002)			RG+de novo
CI009	Gaertnera obovata Baker	Rubiaceae (Rubioideae)	Gaertnereae	Razafimandimbison et al. 980 (S)	2011	Madagascar	RG+de novo
	Gaertnera rotundifolia Bojer	Rubiaceae (Rubioideae)	Gaertnereae	Antonelli et al. (2021)/PAFTOL			RG-only
CA030	Pagamea capitata Benth.	Rubiaceae (Rubioideae)	Gaertnereae	Pipoly 9176 (MEXU)	1986	Guyana	RG+de novo
AP063	Otiophora caerulea (Hiern) Bullock	Rubiaceae (Rubioideae)	Knoxieae	Dessein 367 (BR)	2004	Zambia	RG+de novo
AI014	Chamaepentas hindsioides (K.Schum.) Kårehed & B.Bremer	Rubiaceae (Rubioideae)	Knoxieae	Iversen et al. 85101 (UPS)	1985	Tanzania	de novo
DE064	Triainolepis xerophila (Bremek.) Kårehed & B.Bremer	Rubiaceae (Rubioideae)	Knoxieae	Thureborn et al. 11 (S)	2017	Madagascar	RG+de novo
CQ058	Lasianthus Jack	Rubiaceae (Rubioideae)	Lasiantheae	Razafimandimbison et al. 718 (S)	2009	Vietnam	RG-only
CH072	Lasianthus strigosus Wight	Rubiaceae (Rubioideae)	Lasiantheae	Bremer & Bremer 3902 (UPS)	1998	Australia	RG+de novo
AZ001	Ronabea latifolia Aubl.	Rubiaceae (Rubioideae)	Lasiantheae	Contreras 9152 (S)		Guatemala	RG+de novo
	Saldinia aegialodes Bremek.	Rubiaceae (Rubioideae)	Lasiantheae	Antonelli et al. (2021)/PAFTOL			RG-only
AA052	Saldinia pallida Bremek.	Rubiaceae (Rubioideae)	Lasiantheae	Bremer et al. 4038-BB38 (UPS)	2000	Madagascar	RG+de novo
BE046	Trichostachys microcarpa K.Schum.	Rubiaceae (Rubioideae)	Lasiantheae	Masens 834 (BR)	1991	Congo	RG+de novo
BZ092	Mitchella repens L.	Rubiaceae (Rubioideae)	Mitchelleae	Atha and Gonzalez 1443a (MEXU)	1997	United States, Texas	NA
AQ075	Appunia guatemalensis Donn.Sm.	Rubiaceae (Rubioideae)	Morindeae	Contreras 8983 (S)	1969	Guatemala	RG+de novo
	Coelospermum paniculatum F.Muell.	Rubiaceae (Rubioideae)	Morindeae	Antonelli et al. (2021)/PAFTOL			RG-only
BZ099	Gynochthodes officinalis (F.C.How) Razafim. & B.Bremer	Rubiaceae (Rubioideae)	Morindeae	Krüger et al. 9 (S)	2009	Vietnam	RG+de novo
CV018	Morinda citrifolia L.	Rubiaceae (Rubioideae)	Morindeae	Razafimandimbison 1212a (S)	2013	Madagascar	de novo
AX046	Lerchea bracteata Valeton	Rubiaceae (Rubioideae)	Ophiorrhizeae	Axelius 343 (S)	1983	Indonesia, Sumatra	RG+de novo
	Kajewskiella trichantha Merr. & L.M.Perry	Rubiaceae (Rubioideae)	Ophiorrhizeae	Antonelli et al. (2021)/PAFTOL			RG-only
CH086	Neurocalyx zeylanicus Hook.	Rubiaceae (Rubioideae)	Ophiorrhizeae	Bremer & Bremer 937 (S)	1977	Sri Lanka	RG+de novo
CY100	Ophiorrhiza darwinii Razafim. & Rydin	Rubiaceae (Rubioideae)	Ophiorrhizeae	Swenson et al. 1411 (S)	2013	Vietnam	RG+de novo
CZ012	Ophiorrhiza mungos L.	Rubiaceae (Rubioideae)	Ophiorrhizeae	USA Typhus Commission 525 (S)			RG+de novo
	Ophiorrhiza winkleri Valeton	Rubiaceae (Rubioideae)	Ophiorrhizeae	Antonelli et al. (2021)/PAFTOL			RG-only
	Paederia thouarsiana Baill.	Rubiaceae (Rubioideae)	Paederieae	Antonelli et al. (2021)/PAFTOL			RG+de novo
P0085	Leptodermis potaninii Batalin	Rubiaceae (Rubioideae)	Paederieae	Andreasen 230 (UPS)	1993	Cult. Quarryhill Bot. Gard.	RG+de novo
CA022	Paederia ciliata (Bartl. ex DC.) Standl.	Rubiaceae (Rubioideae)	Paederieae	Campos et al. 4919 (MEXU)	1992	Mexico	RG+de novo
BX093	Pseudopyxis heterophylla (Miq.) Maxim.	Rubiaceae (Rubioideae)	Paederieae	K.Å. Dahlstrand s.n. (GB)	1950	Japan	RG+de novo
C0005	Serissa foetida (L.f.) Lam.	Rubiaceae (Rubioideae)	Paederieae	Bremer 2735 (UPS)	1988	Cult. in Kew Bot. Gard.	RG+de novo
B0110	Spermadictyon suaveolens Roxb.	Rubiaceae (Rubioideae)	Paederieae	Bremer 3133 (UPS)	1988	Cult. in Paris Bot. Gard.	RG+de novo
AE034	Rudgea recurva Müll.Arg.	Rubiaceae (Rubioideae)	Palicoureeae	Pirani et al. 4899 (SPF)	2001	Brazil	RG+de novo
BH070	Palicourea alpina (Sw.) DC.	Rubiaceae (Rubioideae)	Palicoureeae	Rova 2246 (GB)	1995	Cuba	RG+de novo
	Palicourea nitidella (Müll.Arg.) Standl.	Rubiaceae (Rubioideae)	Palicoureeae	Antonelli et al. (2021)/PAFTOL			RG-only

S1 Table. Taxa, voucher/source, ENA/GenBank, assembly, and sequencing information for sequences used in this study.

Taxa with fully assembled plastomes are indicated in grey.

Lab ID	Species	Family (Subfamily)	Tribe (Rubiaceae only)	Collection/Source	Year	Collection locality	Assembly strategy*
CM050	Puffia gerrardii (Baker) Razafim. & B.Bremer	Rubiaceae (Rubioideae)	Palicoureeae	Razafimandimbison et al. 1244 (S)	2013	Madagascar	RG+de novo
	Perama dichotoma Poepp.	Rubiaceae (Rubioideae)	Perameae	Antonelli et al. (2021)/PAFTOL			RG-only
AM028	Perama hirsuta Aubl.	Rubiaceae (Rubioideae)	Perameae	Andersson et al. 1990 (GB)		French Guiana	de novo
CA031	Prismatomeris fragrans E.T.Geddes	Rubiaceae (Rubioideae)	Prismatomerideae	Kainulainen et al. 39 (S)	2009	Vietnam	NA
	Prismatomeris Thwaites	Rubiaceae (Rubioideae)	Prismatomerideae	Antonelli et al. (2021)/PAFTOL			RG-only
CB078	Rennellia subsessilis (King & Gamble) Razafim. & Rydin	Rubiaceae (Rubioideae)	Prismatomerideae	Y.W. Low & Wong LYW 359 (KLU)		Malaysia	RG+de novo
AF076	Psychotria ankarensis (Bremek.) Razafim. & B.Bremer	Rubiaceae (Rubioideae)	Psychotrieae	Razafimandimbison et al. 405 (UPS)	2002	Madagascar	de novo
AG024	Psychotria mahonii C.H.Wright	Rubiaceae (Rubioideae)	Psychotrieae	Luke 8370 (UPS)	2002	Kenya	RG-only
CL028	Calycosia lageniformis (Gillespie) A.C.Sm.	Rubiaceae (Rubioideae)	Psychotrieae	Callmander et al. 962 (S)		Fiji	RG+de novo
	Calycosia petiolata A.Gray	Rubiaceae (Rubioideae)	Psychotrieae	Antonelli et al. (2021)/PAFTOL			RG-only
	Chaetostachydium barbatum Ridsdale	Rubiaceae (Rubioideae)	Psychotrieae	Antonelli et al. (2021)/PAFTOL			RG-only
	Dolianthus montiswilhelmii (P.Royen) A.P.Davis	Rubiaceae (Rubioideae)	Psychotrieae	Antonelli et al. (2021)/PAFTOL			RG+de novo
	Psychotria pandurata Verdc.	Rubiaceae (Rubioideae)	Psychotrieae	Antonelli et al. (2021)/PAFTOL			RG-only
	Plocama calabrica (L.f.) M.Backlund & Thulin	Rubiaceae (Rubioideae)	Putorieae	Antonelli et al. (2021)/PAFTOL			RG-only
AH008	Plocama tinctoria (Balf.f.) M.Backlund & Thulin	Rubiaceae (Rubioideae)	Putorieae	Thulin 10946 (UPS)	2002	Somalia	RG+de novo
AH009	Plocama dubia (Aitch. & Hemsl.) N. Backlund & Thulin	Rubiaceae (Rubioideae)	Putorieae	Rafei & Zangooei 25651 (FUHM)			RG+de novo
	Rubia peregrina L.	Rubiaceae (Rubioideae)	Rubieae	Antonelli et al. (2021)/PAFTOL			RG-only
AG064	Sherardia arvensis L.	Rubiaceae (Rubioideae)	Rubieae	Andreasen 345 (SBT)	2002	Cult. in Bergius Bot. Gard.	RG+de novo
M0004	Didymaea alsinoides (Schltdl. & Cham.) Standl.	Rubiaceae (Rubioideae)	Rubieae	Keller 1901 (CAS)			RG+de novo
DE065	Galium polyacanthum (Baker) Puff	Rubiaceae (Rubioideae)	Rubieae	Thureborn et al. 32 (S)	2017	Madagascar	RG+de novo
T0054	Kelloggia galioides Torr.	Rubiaceae (Rubioideae)	Rubieae	Holmgren et al. 2437 (UPS)	1965	United States, Utah	de novo
BO005	Rubia cordifolia subsp. conotricha (Gand.) Verdc.	Rubiaceae (Rubioideae)	Rubieae	P.A. Luke & W.R.Q Luke 9510 (UPS)	2003	Tanzania	RG+de novo
CH081	Schizocolea linderi (Hutch. & Dalziel) Bremek.	Rubiaceae (Rubioideae)	Schizocoleeae	Adam 20116 (UPS)	1964	Liberia	NA
BZ091	Lecananthus erubescens Jack	Rubiaceae (Rubioideae)	Schradereae	Van Niel 3406 (L)		Borneo, Indonesia	RG+de novo
CX040	Schradera nervulosa (Stapf) Puff, R.Buchner & Greimler	Rubiaceae (Rubioideae)	Schradereae	Puff 961216-2/2 (WU)		Borneo (Sabah)	RG+de novo
CA038	Schradera rotundata Standl. ex Steyerm.	Rubiaceae (Rubioideae)	Schradereae	Gentry 5641 (S)		Panama	de novo
CM080	Seychellea sechellarum (Baker) Razafim., Kainul. & Rydin	Rubiaceae (Rubioideae)	Seychelleeae	C. Morel 57a (SEY)		Seychelles	RG+de novo
CG025	Diodella sarmentosa (Sw.) Bacigalupo & E. L. Cabral	Rubiaceae (Rubioideae)	Spermacoceae	Bremer et al. 5229 (S)	2008	Madagascar	RG+de novo
BZ040	Exallage chrysotricha (Palib.) Neupane & N.Wikstr.	Rubiaceae (Rubioideae)	Spermacoceae	Lin Qinzhong 2004180 (MO)	2004	China	RG+de novo
DE079	Oldenlandia herbacea (L.) Roxb.	Rubiaceae (Rubioideae)	Spermacoceae	Thureborn et al. 27 (S)	2017	Madagacar	de novo
	Spermacoce L.	Rubiaceae (Rubioideae)	Spermacoceae	Antonelli et al. (2021)/PAFTOL			RG-only
	Theligonum cynocrambe L.	Rubiaceae (Rubioideae)	Theligoneae	Antonelli et al. (2021)/PAFTOL			RG+de novo
BV076	Theligonum japonicum Ôkubo & Makino	Rubiaceae (Rubioideae)	Theligoneae	Togashi 6807 (P)	1968	Japan	RG+de novo
AX027	Amphidasya longicalycina (Dwyer) C.M.Taylor	Rubiaceae (Rubioideae)	Urophylleae	Huber 2963 (CR)		Costa Rica	RG+de novo
AS084	Temnopteryx sericea Hook.f.	Rubiaceae (Rubioideae)	Urophylleae	Tabak 999 (WAG)		Gabon	RG+de novo
	Urophyllum cyphandrum Stapf	Rubiaceae (Rubioideae)	Urophylleae	Antonelli et al. (2021)/PAFTOL			RG-only
AY100	Raritebe palicoureoides Wernham	Rubiaceae (Rubioideae)	Urophylleae	Antonio 1697 (AAU)		Panama	de novo
BA031	Urophyllum arboreum (Reinw. ex Blume) Korth.	Rubiaceae (Rubioideae)	Urophylleae	Boeea 7887 (S)	1935	Sumatra	RG-only

*See the main text for details

**ENA accessions (beginning with ERR) refer to raw sequence reads. The respective assembled plastomes of these samples are available in the Dryad Digital Repository (https://doi.org/10.5061/dryad.mpg4f4r67)

S1 Table. Taxa, voucher/source, ENA/GenBank, assembly, and sequencing information for sequences used in this study.

Taxa with fully assembled plastomes are indicated in grey.

Lab ID	Species	Family (Subfamily)	Tribe (Rubiaceae only)	Assembly reference seq	Plastome coverage	# Reads after dedupe and trimming	ENA/GenBank accesions**
	Alstonia scholaris (L.) R.Br.	Apocynaceae		MN176280	12.0	3666186	ERR5034019
	Chironia baccifera L.	Gentianaceae		ON641347	10.4	1484028	ERR5033620
	Geniostoma borbonicum (Lam.) Spreng.	Loganiaceae		MT471262	72.2	5420570	ERR5034022
BD003	Acranthera grandiflora Bedd.	Rubiaceae	Coptosapelteae	KY378704	41.4	9044646	ERR9883540
AY008	Coptosapelta diffusa (Champ. ex Benth.) Steenis	Rubiaceae	Coptosapelteae	KY378704	180.9	8909362	ERR9883544
AZ040	Luculia pinceana Hook.	Rubiaceae	Luculieae	NA	134.5	11582072	ERR9883503
DE068	Cinchona calisaya Wedd.	Rubiaceae (Cinchonoideae)	Cinchoneae	MZ151891	224.2	22815466	ERR9883472
CC071	Deppea grandiflora Schltdl.	Rubiaceae (Cinchonoideae)	Hamelieae	KY378675	22.7	11600302	ERR9883535
DE066	Paracorynanthe antankarana Capuron ex J.-F.Leroy	Rubiaceae (Cinchonoideae)	Hymenodictyeae	KY378679	63.8	29947254	ERR9883470+ERR9883475
	Corynanthe pachyceras K.Schum.	Rubiaceae (Cinchonoideae)	Naucleeae	KY378678	3.7	2323758	ERR5033631
AG098	Rondeletia intermixta Britton	Rubiaceae (Cinchonoideae)	Rondeletieae	KY378681	80.8	19596792	ERR9883534
AP035	Airosperma vanuense S.P.Darwin	Rubiaceae (Ixoroideae)	Airospermeae	KY348840	60.8	9756614	ERR9883530
	Razafimandimbisonia minor (Baill.) Kainul. & B.Bremer	Rubiaceae (Ixoroideae)	Alberteae	KY348839	7.9	1912346	ERR5033626
BE034	Wendlandia dasythyrsa Miq.	Rubiaceae (Ixoroideae)	Augusteae	KY492076	100.5	42365064	ERR9883548
BD032	Alseis lugonis L.Andersson	Rubiaceae (Ixoroideae)	Condamineeae	NC_036300	29.5	12403114	ERR9883484
	Emmenopterys henryi Oliv.	Rubiaceae (Ixoroideae)	Condamineeae	NC_036300	10.5	1894852	ERR5033635
CX014	Ixora javanica (Blume) DC.	Rubiaceae (Ixoroideae)	Ixoreae	KY378663	67.1	24509876	ERR9883554
	Jackiopsis ornata (Wall.) Ridsdale	Rubiaceae (Ixoroideae)	Jackieae	KY378669	4.3	2271120	ERR5033822
	Heinsia crinita (Wennberg) G.Taylor	Rubiaceae (Ixoroideae)	Mussaendeae	KY348834	3.3	978442	ERR5033638
AV015	Molopanthera paniculata Turcz.	Rubiaceae (Ixoroideae)	Posoquerieae	NA	74.0	21299400	ERR9883549
CG080	Sabicea marojejyensis Razafim. & J.S.Mill.	Rubiaceae (Ixoroideae)	Sabiceeae	NA	357.2	18732102	ERR9883531
	Cyclophyllum barbatum (G.Forst.) N.Hallé & J.Florence	Rubiaceae (Ixoroideae)	Vanguerieae	KY378666	57.3	10233994	ERR5034026
CA098	Glionnetia sericea (Baker) Tirveng.	Rubiaceae (Ixoroideae)		NA	111.4	8109216	ERR9883500
DE070	Anthospermum thymoides Baker	Rubiaceae (Rubioideae)	Anthospermeae	CY051: this study	26.8	12886590	ERR9883532
DE067	Carpacoce spermacocea (Rchb. ex Spreng.) Sond.	Rubiaceae (Rubioideae)	Anthospermeae	CY051: this study	913.3	23868312	ERR9883471
	Coprosma longifolia A.Gray	Rubiaceae (Rubioideae)	Anthospermeae	CY003: this study	29.7	3417710	ERR5034025
CY012	Coprosma rhamnoides A.Cunn.	Rubiaceae (Rubioideae)	Anthospermeae	CY003: this study	144.9	13855942	ERR9883542
DE081	Durringtonia paludosa R.J.F.Hend. & Guymer	Rubiaceae (Rubioideae)	Anthospermeae	CY003: this study	36.0	9797814	ERR9883564
CX098	Galopina aspera (Eckl. & Zeyh.) Walp.	Rubiaceae (Rubioideae)	Anthospermeae	CY051: this study	46.6	8056560	ERR9883565
CX099	Leptostigma pilosum (Benth.) Fosberg	Rubiaceae (Rubioideae)	Anthospermeae	CY003: this study	200.0	19283612	ERR9883489+ERR11475413
CY003	Nertera dichondrifolia (A.Cunn.) Hook.f.	Rubiaceae (Rubioideae)	Anthospermeae	NA	108.9	9800008	ERR9883499
DE075	Normandia neocaledonica Hook.f.	Rubiaceae (Rubioideae)	Anthospermeae	CY003: this study	206.9	13472450	ERR9883512
CY089	Opercularia volubilis R.Br. ex Benth.	Rubiaceae (Rubioideae)	Anthospermeae	NA	268.1	11482826	ERR9883537
CY051	Phyllis nobla L.	Rubiaceae (Rubioideae)	Anthospermeae	NA	1051.4	14189504	ERR9883493
DE074	Pomax umbellata (Gaertn.) Sol. ex A.Rich.	Rubiaceae (Rubioideae)	Anthospermeae	CY003: this study	360.2	16524524	ERR9883546
BL040	Argostemma elatostemma Hook.f.	Rubiaceae (Rubioideae)	Argostemmateae	CD007: this study	73.8	12664432	ERR9883520
BU095	Clarkella nana (Edgew.) Hook.f.	Rubiaceae (Rubioideae)	Argostemmateae	CD007: this study	11.7	6379438	ERR9883563
CH091	Mouretia larsenii Tange	Rubiaceae (Rubioideae)	Argostemmateae	NA	171.9	18240426	ERR9883550
CD007	Mycetia bracteata Hutch.	Rubiaceae (Rubioideae)	Argostemmateae	NA	112.1	10711816	ERR9883494
	Mycetia Reinw.	Rubiaceae (Rubioideae)	Argostemmateae	CD007: this study	15.9	1893208	ERR5084289
BK078	Neohymenopogon parasiticus (Wall.) Bennet	Rubiaceae (Rubioideae)	Argostemmateae	CD007: this study	100.1	7139796	ERR9883558
BD002	Colletoecema dewevrei (De Wild.) E.M.A.Petit	Rubiaceae (Rubioideae)	Colletoecemateae	NA		NA	KY378707
BA061	Coccocypselum condalia Pers.	Rubiaceae (Rubioideae)	Coussareeae	KY378701 + AY100: this study	376.5	12339184	ERR9883533

S1 Table. Taxa, voucher/source, ENA/GenBank, assembly, and sequencing information for sequences used in this study.
Taxa with fully assembled plastomes are indicated in grey.

Lab ID	Species	Family (Subfamily)	Tribe (Rubiaceae only)	Assembly reference seq	Plastome coverage	# Reads after dedupe and trimming	ENA/GenBank accesions**
DA042	Cruckshanksia pumila Clos in C.Gay	Rubiaceae (Rubioideae)	Coussareeae	KY378701 + AY100: this study	2259.7	17990498	ERR9883536
DB007	Faramea multiflora A.Rich. ex DC.	Rubiaceae (Rubioideae)	Coussareeae	KY378701 + AY100: this study	167.6	27105744	ERR9883469+ERR9883477
	Craterispermum Benth. 2	Rubiaceae (Rubioideae)	Craterispermeae	KY378698	14.5	2256818	ERR5084275
CK013	Craterispermum Benth. 1	Rubiaceae (Rubioideae)	Craterispermeae	KY378698	123.5	7060026	ERR9883485
CL038	Craterispermum schweinfurthii Hiern	Rubiaceae (Rubioideae)	Craterispermeae	KY378698	95.3	7542388	ERR9883498
BZ084	Cyanoneuron cyaneum (Hallier f.) Tange	Rubiaceae (Rubioideae)	Cyanoneuroneae	MN883829	89.2	12785706	ERR9883481
	Cyanoneuron pedunculatum Tange	Rubiaceae (Rubioideae)	Cyanoneuroneae	BZ084: this study	0.5	676640	ERR5034803
DB070	Danais nigra Homolle	Rubiaceae (Rubioideae)	Danaideae	DB077:this study	171.0	18501058	ERR9883478
DB077	Payera decaryi (Homolle) Buchner & Puff	Rubiaceae (Rubioideae)	Danaideae	NA	148.3	26955428	ERR9883539
DC094	Schismatoclada marojejyensis Humbert	Rubiaceae (Rubioideae)	Danaideae	NA	479.4	22354314	ERR9883567
BC005	Dunnia sinensis Tutcher	Rubiaceae (Rubioideae)	Dunnieae	MN883829	509.7	14233770	ERR9883505
CI009	Gaertnera obovata Baker	Rubiaceae (Rubioideae)	Gaertnereae	KY378695	242.3	5111686	ERR9883479
	Gaertnera rotundifolia Bojer	Rubiaceae (Rubioideae)	Gaertnereae	KY378695	5.2	842634	ERR5033654
CA030	Pagamea capitata Benth.	Rubiaceae (Rubioideae)	Gaertnereae	KY378695	24.7	10429052	ERR9883502
AP063	Otiophora caerulea (Hiern) Bullock	Rubiaceae (Rubioideae)	Knoxieae	AI014: this study	116.2	8571588	ERR9883525
AI014	Chamaepentas hindsioides (K.Schum.) Kårehed & B.Bremer	Rubiaceae (Rubioideae)	Knoxieae	NA	314.4	10619468	ERR9883496
DE064	Triainolepis xerophila (Bremek.) Kårehed & B.Bremer	Rubiaceae (Rubioideae)	Knoxieae	AI014: this study	983.5	50239610	ERR9883541
CQ058	Lasianthus Jack	Rubiaceae (Rubioideae)	Lasiantheae	CH072: this study	41.4	16361860	ERR9883556
CH072	Lasianthus strigosus Wight	Rubiaceae (Rubioideae)	Lasiantheae	KY378708	86.0	10421850	ERR9883504
AZ001	Ronabea latifolia Aubl.	Rubiaceae (Rubioideae)	Lasiantheae	CH072: this study	308.4	9834184	ERR9883501
	Saldinia aegialodes Bremek.	Rubiaceae (Rubioideae)	Lasiantheae	AA052: this study	10.6	1649566	ERR5033656
AA052	Saldinia pallida Bremek.	Rubiaceae (Rubioideae)	Lasiantheae	AY100: this study	3841.1	61673872	ERR9883473+ERR9883476
BE046	Trichostachys microcarpa K.Schum.	Rubiaceae (Rubioideae)	Lasiantheae	AY100: this study	109.1	11768826	ERR9883545
BZ092	Mitchella repens L.	Rubiaceae (Rubioideae)	Mitchelleae	NA		NA	KY378710
AQ075	Appunia guatemalensis Donn.Sm.	Rubiaceae (Rubioideae)	Morindeae	CV018: this study	88.8	5879036	ERR9883516
	Coelospermum paniculatum F.Muell.	Rubiaceae (Rubioideae)	Morindeae	CV018: this study	18.7	1270746	ERR5034257
BZ099	Gynochthodes officinalis (F.C.How) Razafim. & B.Bremer	Rubiaceae (Rubioideae)	Morindeae	CV018: this study	178.2	19554914	ERR9883527
CV018	Morinda citrifolia L.	Rubiaceae (Rubioideae)	Morindeae	NA	693.4	23023506	ERR9883553
AX046	Lerchea bracteata Valeton	Rubiaceae (Rubioideae)	Ophiorrhizeae	MW528277	127.6	28155166	ERR9883566
	Kajewskiella trichantha Merr. & L.M.Perry	Rubiaceae (Rubioideae)	Ophiorrhizeae	MW528277	3.5	424886	ERR5034845
CH086	Neurocalyx zeylanicus Hook.	Rubiaceae (Rubioideae)	Ophiorrhizeae	MW528277	147.7	16014084	ERR9883555
CY100	Ophiorrhiza darwinii Razafim. & Rydin	Rubiaceae (Rubioideae)	Ophiorrhizeae	MW528277	69.9	13073088	ERR9883497
CZ012	Ophiorrhiza mungos L.	Rubiaceae (Rubioideae)	Ophiorrhizeae	MW528277	177.9	10451454	ERR9883526
	Ophiorrhiza winkleri Valeton	Rubiaceae (Rubioideae)	Ophiorrhizeae	MW528277	5.1	852822	ERR5033642
	Paederia thouarsiana Baill.	Rubiaceae (Rubioideae)	Paederieae	NC_049155	34.4	2389358	ERR5033643
P0085	Leptodermis potaninii Batalin	Rubiaceae (Rubioideae)	Paederieae	NC_049155	200.1	15133514	ERR9883547
CA022	Paederia ciliata (Bartl. ex DC.) Standl.	Rubiaceae (Rubioideae)	Paederieae	NC_049155	92.0	12507838	ERR9883513
BX093	Pseudopyxis heterophylla (Miq.) Maxim.	Rubiaceae (Rubioideae)	Paederieae	NC_049155	49.4	22257894	ERR9883524
C0005	Serissa foetida (L.f.) Lam.	Rubiaceae (Rubioideae)	Paederieae	NC_049155	180.8	15697518	ERR9883487
B0110	Spermadictyon suaveolens Roxb.	Rubiaceae (Rubioideae)	Paederieae	NC_049155	183.4	12712938	ERR9883508
AE034	Rudgea recurva Müll.Arg.	Rubiaceae (Rubioideae)	Palicoureeae	KY378697	22.1	7703636	ERR9883488
BH070	Palicourea alpina (Sw.) DC.	Rubiaceae (Rubioideae)	Palicoureeae	KY378697	42.4	17823444	ERR9883557
	Palicourea nitidella (Müll.Arg.) Standl.	Rubiaceae (Rubioideae)	Palicoureeae	KY378697	7.0	954302	ERR5033644

S1 Table. Taxa, voucher/source, ENA/GenBank, assembly, and sequencing information for sequences used in this study.

Taxa with fully assembled plastomes are indicated in grey.

Lab ID	Species	Family (Subfamily)	Tribe (Rubiaceae only)	Assembly reference seq	Plastome coverage	# Reads after dedupe and trimming	ENA/GenBank accesions**
CM050	Puffia gerrardii (Baker) Razafim. & B.Bremer	Rubiaceae (Rubioideae)	Palicoureeae	KY378697	53.4	6652086	ERR9883480
	Perama dichotoma Poepp.	Rubiaceae (Rubioideae)	Perameae	AM028: this study	9.9	1938690	ERR5033646
AM028	Perama hirsuta Aubl.	Rubiaceae (Rubioideae)	Perameae	NA	284.7	24301482	ERR9883561
CA031	Prismatomeris fragrans E.T.Geddes	Rubiaceae (Rubioideae)	Prismatomerideae	NA	NA	NA	KY378699
	Prismatomeris Thwaites	Rubiaceae (Rubioideae)	Prismatomerideae	KY378699	6.3	1217278	ERR5084277
CB078	Rennellia subsessilis (King & Gamble) Razafim. & Rydin	Rubiaceae (Rubioideae)	Prismatomerideae	KY378699	367.2	16352526	ERR9883521
AF076	Psychotria ankarensis (Bremek.) Razafim. & B.Bremer	Rubiaceae (Rubioideae)	Psychotrieae	NA	99.6	3212850	ERR9883486
AG024	Psychotria mahonii C.H.Wright	Rubiaceae (Rubioideae)	Psychotrieae	KY378696	33.4	5238420	ERR9883483
CL028	Calycosia lageniformis (Gillespie) A.C.Sm.	Rubiaceae (Rubioideae)	Psychotrieae	AF076:this study	103.6	5095646	ERR9883559
	Calycosia petiolata A.Gray	Rubiaceae (Rubioideae)	Psychotrieae	CL028: this study	5.6	1412462	ERR5034836
	Chaetostachydium barbatum Ridsdale	Rubiaceae (Rubioideae)	Psychotrieae	CL028: this study	2.0	670082	ERR5034846
	Dolianthus montiswilhelmii (P.Royen) A.P.Davis	Rubiaceae (Rubioideae)	Psychotrieae	AF076:this study	28.9	3568582	ERR5034835
	Psychotria pandurata Verdc.	Rubiaceae (Rubioideae)	Psychotrieae	KY378696	1.5	778050	ERR5033649
	Plocama calabrica (L.f.) M.Backlund & Thulin	Rubiaceae (Rubioideae)	Putorieae	KY378690	8.2	1461868	ERR5033647
AH008	Plocama tinctoria (Balf.f.) M.Backlund & Thulin	Rubiaceae (Rubioideae)	Putorieae	KY378690	346.4	18586312	ERR9883529
AH009	Plocama dubia (Aitch. & Hemsl.) N. Backlund & Thulin	Rubiaceae (Rubioideae)	Putorieae	KY378690	58.4	6150778	ERR9883507
	Rubia peregrina L.	Rubiaceae (Rubioideae)	Rubieae	NC_047470	4.2	3603986	ERR5033650
AG064	Sherardia arvensis L.	Rubiaceae (Rubioideae)	Rubieae	NC_047470	389.1	21195568	ERR9883522
M0004	Didymaea alsinoides (Schltdl. & Cham.) Standl.	Rubiaceae (Rubioideae)	Rubieae	NC_047470	92.0	10419852	ERR9883528
DE065	Galium polyacanthum (Baker) Puff	Rubiaceae (Rubioideae)	Rubieae	NC_047470	84.6	6173124	ERR9883538
T0054	Kelloggia galioides Torr.	Rubiaceae (Rubioideae)	Rubieae	NA	2114.7	38841036	ERR9883509+ERR9883560
BO005	Rubia cordifolia subsp. conotricha (Gand.) Verdc.	Rubiaceae (Rubioideae)	Rubieae	NC_047470	302.4	32273554	ERR9883467+ERR9883474
CH081	Schizocolea linderi (Hutch. & Dalziel) Bremek.	Rubiaceae (Rubioideae)	Schizocoleeae	NA		NA	KY378700
BZ091	Lecananthus erubescens Jack	Rubiaceae (Rubioideae)	Schradereae	CA038: this study	55.8	11232010	ERR9883543
CX040	Schradera nervulosa (Stapf) Puff, R.Buchner & Greimler	Rubiaceae (Rubioideae)	Schradereae	CA038: this study	212.2	20908840	ERR9883523
CA038	Schradera rotundata Standl. ex Steyerm.	Rubiaceae (Rubioideae)	Schradereae	NA	329.0	8213208	ERR9883510
CM080	Seychellea sechellarum (Baker) Razafim., Kainul. & Rydin	Rubiaceae (Rubioideae)	Seychelleeae	KY378707	74.3	7073510	ERR9883491
CG025	Diodella sarmentosa (Sw.) Bacigalupo & E. L. Cabral	Rubiaceae (Rubioideae)	Spermacoceae	DE079:this study	81.1	7522408	ERR9883492
BZ040	Exallage chrysotricha (Palib.) Neupane & N.Wikstr.	Rubiaceae (Rubioideae)	Spermacoceae	DE079:this study	84.7	8374914	ERR9883482
DE079	Oldenlandia herbacea (L.) Roxb.	Rubiaceae (Rubioideae)	Spermacoceae	NA	1079.9	28408956	ERR9883552
	Spermacoce L.	Rubiaceae (Rubioideae)	Spermacoceae	DE079:this study	9.6	1180896	ERR5084278
	Theligonum cynocrambe L.	Rubiaceae (Rubioideae)	Theligoneae	KY378688	78.1	5963764	ERR5033810
BV076	Theligonum japonicum Ôkubo & Makino	Rubiaceae (Rubioideae)	Theligoneae	KY378688	303.0	57226818	ERR9883515+ERR9883518
AX027	Amphidasya longicalycina (Dwyer) C.M.Taylor	Rubiaceae (Rubioideae)	Urophylleae	AY100: this study	63.2	7053006	ERR9883495
AS084	Temnopteryx sericea Hook.f.	Rubiaceae (Rubioideae)	Urophylleae	AY100: this study	70.8	9626114	ERR9883562
	Urophyllum cyphandrum Stapf	Rubiaceae (Rubioideae)	Urophylleae	AY100: this study	4.0	1574358	ERR5033613
AY100	Raritebe palicoureoides Wernham	Rubiaceae (Rubioideae)	Urophylleae	NA	269.7	9986836	ERR9883517
BA031	Urophyllum arboreum (Reinw. ex Blume) Korth.	Rubiaceae (Rubioideae)	Urophylleae	AY100: this study	11.3	3468372	ERR9883490

*See the main text for details

**ENA accessions (beginning with ERR) refer to raw sequence reads. The respective assembled plastomes of these samples are available in the Dryad Digital Repository (https://doi.org/10.5061/dryad.mpg4f4r67)
