# Supplementary material for: Plastid phylogenomics and cytonuclear discordance in Rubioideae, Rubiaceae
Source: PLoS One. 2024 May 20;19(5):e0302365. doi: 10.1371/journal.pone.0302365 (PMC11104678; doi:10.1371/journal.pone.0302365)
Supplement: S1 Fig — (PDF) [file pone.0302365.s002.pdf]

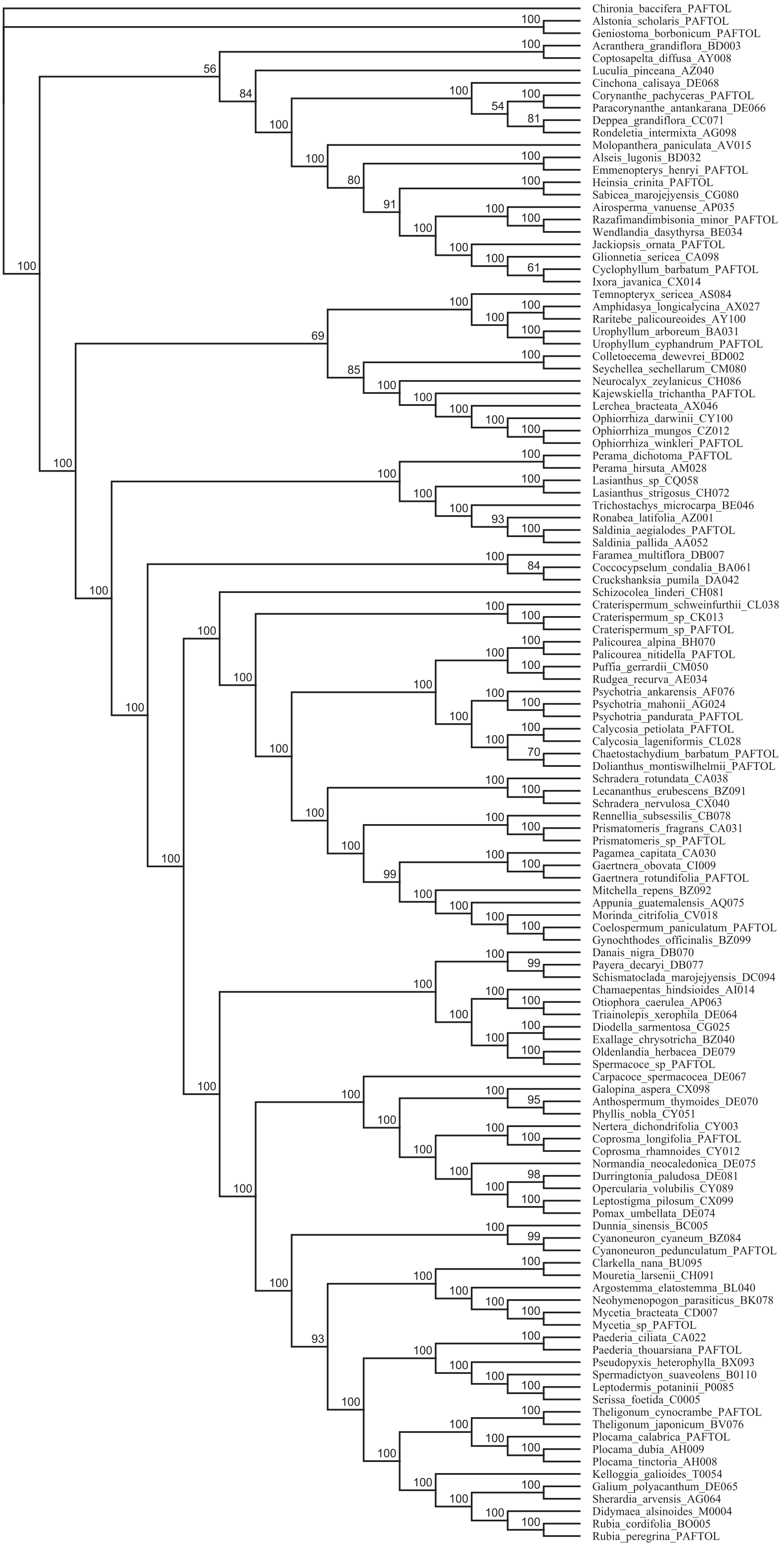

S1 Fig. Plastome phylogeny inferred from maximum likelihood analysis of the RY-coded version of alignment 1. The four nucleotides were recoded into two states (purines and pyrimidines). Alignment 1 was untrimmed except for removal of autapomorphies (columns where more than 99% of the samples had a gap). Numbers above branches indicate bootstrap support values.
