# Supplementary material for: Plastid phylogenomics and cytonuclear discordance in Rubioideae, Rubiaceae
Source: PLoS One. 2024 May 20;19(5):e0302365. doi: 10.1371/journal.pone.0302365 (PMC11104678; doi:10.1371/journal.pone.0302365)
Supplement: S2 Fig — (PDF) [file pone.0302365.s003.pdf]

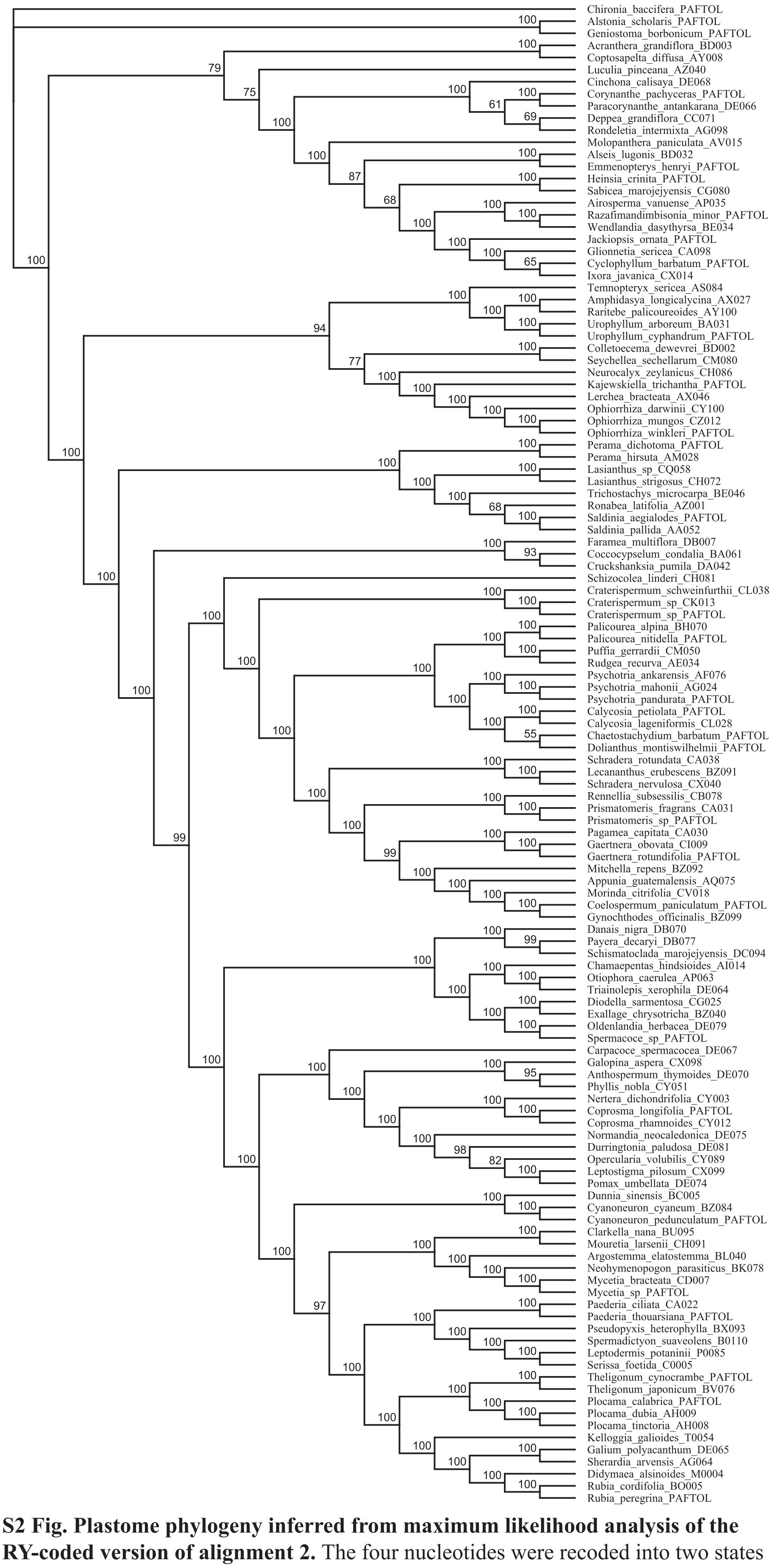

S2 Fig. Plastome phylogeny inferred from maximum likelihood analysis of the RY-coded version of alignment 2. The four nucleotides were recoded into two states (purines and pyrimidines). Alignment 2 was trimmed using trimAl. Numbers above branches indicate bootstrap support values.
